# Robert C. Dunnell's *Systematics in prehistory* at 50

**DOI:** 10.1017/ehs.2022.18

**Published:** 2022-04-27

**Authors:** Felix Riede, Astolfo Araujo, Ben Marwick

**Affiliations:** 1 Department of Archaeology and Heritage Studies, Aarhus University, Moesgård Allé 20, 8270 Højbjerg, Denmark; 2 Museum of Archaeology and Ethnology, University of São Paulo, Brazil; 3 Department of Anthropology, University of Washington, Seattle, WA, USA

**Keywords:** Systematics, evolutionary archaeology, cultural phylogenetics, classification, Robert C. Dunnell

## Abstract

The year 2021 marked the fiftieth anniversary of the publication of Robert C. Dunnell's (Free Press, 1971) diminutive yet dense *Systematics in prehistory*. At the height of the debate between Culture History and New Archaeology, Dunnell's work sought to address a more fundamental issue that was and still is relevant to all branches of prehistoric archaeology, and especially to the study of the Palaeolithic: systematics. Dunnell himself was notorious and controversial, but the importance of his work remains underappreciated. Like other precocious works of that tumultuous time, *Systematics in prehistory* today remains absent from most course reading lists and gathers dust on library shelves. In this contribution we argue for a greater appreciation of its as yet unfulfilled conceptual and analytical promise. In particular, we briefly chart its somewhat delayed impact via evolutionary archaeology, including how it has also influenced non-Anglophone traditions, especially in South America. The obstinate persistence of classification issues in palaeoanthropology and palaeoarchaeology, we argue, warrants a second look at Dunnell's *Systematics*.

## Graphical abstract

**Figure:**
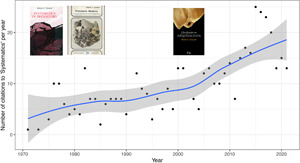

**Social media summary:** Dunnell's iconic *Systematics in prehistory*’ at 50 – a fresh look at the book's legacy, impact and continual relevance

## 1. Introduction

Evolutionary archaeology is concerned with the study of cultural evolution through material culture proxies and commonly over extended timescales not readily accessible by other means. While evolutionary thinking *per se* is not new in the discipline, an operational evolutionary archaeology emerged only recently, and on the back of several major paradigm transitions (Prentiss, 2019). The notion of major paradigm shifts in the history of archaeology is, however, characterized by a great deal of hyperbole and rhetorical manoeuvring. That said, the late 1960s and 1970s do stand out as revolutionary. In the US, the Binfordian juggernaut increasingly hammered away at its culture-historical nemesis, while in the UK, David Clarke's (1968) formidable *Analytical archaeology* was shaking up the establishment with novel concepts and methods as well as incisive rhetoric. The reception and impact of these works have seen a great deal of attention in later years, especially as many researchers working within ecological and evolutionary approaches to past culture change are rediscovering the merits of Clarke's conceptual approach in particular, and Binford's extensive data synthesis (Lycett & Shennan, 2018; Nicholas, 2012). The year 2021 marked the fiftieth anniversary of the publication of Robert C. Dunnell's (1971) diminutive yet dense *Systematics in prehistory*, a volume that is concerned entirely with classification in archaeology. Appearing at the height of the debate between Culture History and New Archaeology, Dunnell's work was no less iconoclastic than that of Binford or Clarke, but it was considerably narrower in its goal of addressing a more fundamental issue that was and still is relevant to all branches of archaeology: systematics.

Dunnell (1942–2010) himself was notorious and controversial, and his *Systematics* was received with mixed reactions by his contemporaries who commented – mostly negatively – both on his style of writing and the book's content (Bayard, 1973; Shenkel, 1973; Spaulding, 1974; Tuggle, 1974). Like other precocious works of that tumultuous time, *Systematics in prehistory* today remains absent from most course reading lists and gathers dust on library shelves. As Lyman has recently shown, systematics takes up little space in contemporary archaeological research or teaching (Lyman, 2021). In striking contrast, biological systematics is a well-developed field with faculty positions, courses and journals dedicated to this fundamental scientific concern. If at least some of the success of Clarke and Binford can be attributed to them boldly tackling exciting and large-scale topics such as migration and adaptation through the introduction of avant-garde terminology, then the corresponding obscurity of Dunnell's *Systematics* can perhaps be attributed to him focusing on an issue that simply seemed too quotidian. Furthermore, archaeology has moved ahead in such a way that leaves some of the key claims *Systematics* exposed as distinct outliers in modern (American) archaeology. For example, his insistence that the exclusive focus of archaeology be the physical traces of pasts human activity, and that archaeology cannot be both a science and a sub-discipline of anthropology, are generally minority positions now, with archaeology continuing to be a sub-field of anthropology in most US universities at least. In countries where archaeology is situated in different institutional contexts, this particular issue is not a major concern. Be it as it may, Dunnell was correct in his axiomatic insistence that rigorous classification comes before any other analysis or interpretation. Without consistent and explicit classification, any scientific discipline will inevitably fail to produce cumulative insights; certainly, evolutionary analysis would hardly be possible without robust classification.

## 2. *Systematics in prehistory* and the emergence of evolutionary archaeology

For Dunnell himself, the soul-searching that began with writing *Systematics in prehistory* led him to discover evolutionary theory. In a series of follow-up papers, he forcefully argued for the benefits of a scientific and Darwinian archaeology (Dunnell, 1980, 1982). While his writing style did not attract many followers, these arguments have since become foundational for the development of evolutionary archaeology, especially in the Americas (O'Brien, 1996). Initially, following the direct lead of Dunnell, this approach was rather narrowly selectionist, treating artefacts as the hard parts of the human phenotype and selection acting on these as the main driver of change (O'Brien & Holland, 1990). Evolutionary archaeology has since become both more plural and more fully aligned with cultural evolutionary thinking in the form of behavioural ecology, dual-inheritance and niche construction theory (Marwick, 2006; Prentiss, 2021; Riede, 2019). Vitally, cultural evolutionary theory and its focus on the transmission of cultural knowledge via various modes of learning has provided the crucial generative mechanism for material culture systematics. In its contemporary form, selection, but also drift and a range of transmission biases, play important roles in explaining culture change.

While Dunnell worked exclusively on the Holocene prehistory of the Americas, the idiosyncrasies of archaeological classification are nowhere more apparent and acute than in the archaeology of human evolutionary deep history: the Palaeolithic. Rooted in French antiquarianism, the development of Palaeolithic systematics, for instance, has been likened to so many ‘accidents of history’ (Clark, 2009), but there are few periods or regions of the Palaeolithic that have not seen debate about the validity or otherwise of their analytical units (Reynolds & Riede, 2019). The use of older typological classifications remains prevalent, despite clear and repeated critiques (e.g. Bisson, 2000). More recently, the analysis of technological traits has supplemented or even eclipsed purely typological approaches. However, theoretical explications of the generative mechanisms, and rigorous comparative systematics backed by transparent and replicable analytics as demanded by Dunnell, remain exceptions rather than the rule (Tostevin, 2013). Today, a great deal of attention is again being paid to systematics in prehistory, and as Barton and Clark (2021) have pointed out, the continuing adherence to outmoded classifications is preventing the exploration of more relevant and pressing research questions. Several researchers are tackling classificatory issues with novel and mostly quantitative means (Grove & Blinkhorn, 2021; Ivanovaitė et al., 2020; Leplongeon et al., 2020). At the same time, however, the topic remains poorly heeded in archaeological pedagogy (Lyman, 2021). Given that cultural evolutionary theory itself teaches us that aspects of culture most easily and rapidly change when scaffolded through active teaching (Riede et al., 2021), we recommend that, after half a century, Dunnell's *Systematics in prehistory* – and with it rigorous and replicable ways of classifying material culture – are placed more abundantly on our curricula, in addition to continuing the ongoing critique and transformation of existing classifications through novel research. Only when the construction and meaning of our analytical units and their relationships among one another are transparent and robust cultural taxonomies are in place can we seriously hope to understand the patterns and processes that have shaped cultural evolution in deep history.

## 3. The contribution of *Systematics*

To survey the contribution that *Systematics in prehistory* has made to evolutionary anthropology, we take inspiration from its concept of statistical clustering as a method for organising variability and recent developments in the statistical analysis of text. We searched for items citing *Systematics in prehistory* on Google Scholar, downloaded all the search result pages (data were collected on July 2021) and extracted bibliographic data from each work citing *Systematics*. The raw data and R code for our analysis of the Google Scholar results, and the R Markdown source document for this paper are openly available online at http://doi.org/10.17605/OSF.IO/JBPFW.

**Figure 1.**
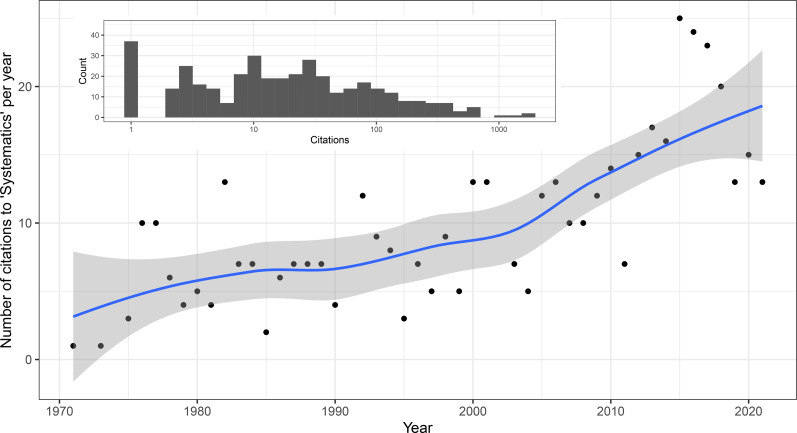
Citations to ‘Systematics’ over time. Inset shows distributions of citations to works citing ‘Systematics’, i.e. the degree to which works citing ‘Systematics’ have themselves been cited. Data collected from Google Scholar in July 2021.

**Figure 2.**
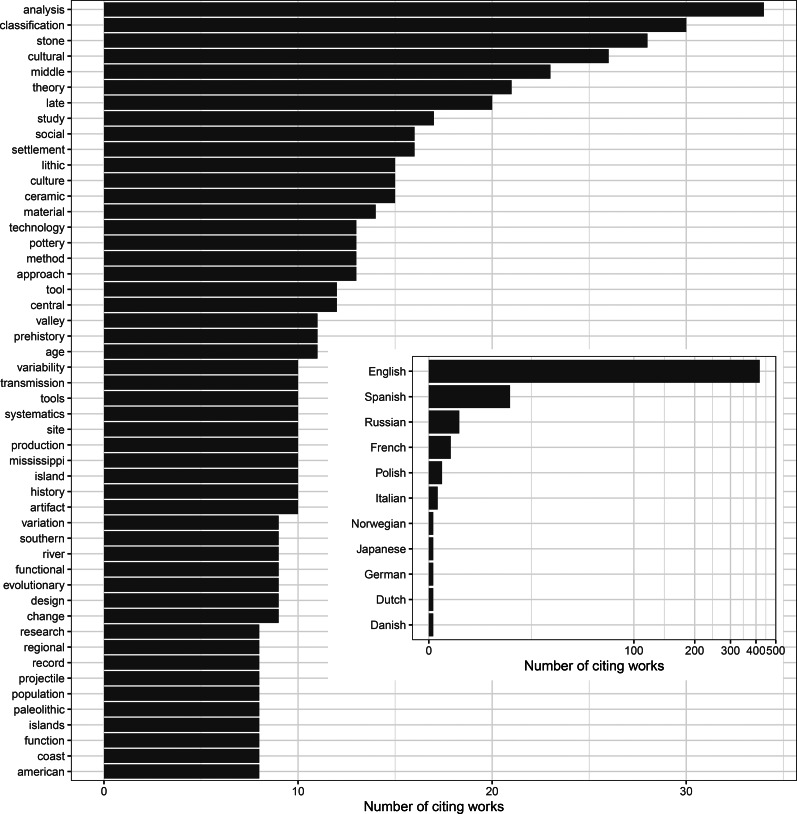
Keywords in titles of works citing ‘Systematics’. Inset shows languages of works citing ‘Systematics’.

We found 475 citations of *Systematics*; for reference, the most highly cited archaeology publication from the same year is Binford's (1971) ‘Mortuary practices: Their study and their potential’, with 1621 citations currently on Google Scholar at the time of our data collection. Citations to *Systematics* have steadily accumulated over time, with a distinct increase in the annual rate of citation in the early 2000s as evolutionary concepts become increasingly integrated into archaeological science (Figure 1). Several works that cite *Systematics* have themselves been very highly cited, with 10 publications receiving over 500 citations, demonstrating its influence over a range of topics including social theory (Shanks & Tilley, 1987), behavioural archaeology (Schiffer, 2016) and lithic analysis (Andrefsky, 1998). Looking at the top 50 words in the titles of the works citing *Systematics*, we can also see that ‘lithics’ and ‘stone’ are prominent, indicating that its contribution was especially noted by archaeologists working on stone artefacts (Figure 2). Pottery is the only other artefact type where analysts substantially engaged with *Systematics*. Notable in the title keywords are concepts relating to evolution, for example, ‘transmission’, ‘variation’ and ‘evolutionary’. Evolution is a very minor theme in *Systematics* itself, but these word frequency data show it has proven to be a foundational text in applications of evolutionary theory to explaining variability in the archaeological record.

Beyond the Anglosphere, we see citations from a small number works in Russian and European languages (Figure 2), but the impact of *Systematics* is most evident in Spanish-language publications – *Systematics* was published in its Spanish translation already in 1977 – by scholars working on South American stone artefact assemblages. These studies have been especially innovative, taking to heart the critique in *Systematics* that archaeological typologies are often intuitive, arbitrary and difficult to replicate by other researchers. Motivated by this critique, scholars such as Marcelo Cardillo, Judith Charlin (Cardillo & Charlin, 2018), Mercedes Okumura (Okumura & Araujo, 2014) and others have conducted pioneering work in the application of geometric morphometrics to stone artefact assemblages in an effort to provide a materialist view of technological variation where the focus is on continuous quantitative phenomena. While geometric morphometrics has been applied by archaeologists to a range of regions and artefact types (including ceramic and metal), what makes this South American work remarkable as part of the legacy of *Systematics* is their exploration of modern phylogenetic comparative methods to model and quantify technological variation and change over space and time (e.g. Cardillo & Alberti, 2015). While the work by Cardillo and colleagues (Cardillo & Alberti, 2015; Cardillo & Charlin, 2018) focuses on phylogenetic signals and material culture diversity across time and space, Okumura and Araujo (2014) are more concerned with the persistence of attributes over long stretches of time. What these works have in common is the use of an explicitly cultural evolutionary rationale. In these studies, the use of paradigmatic classifications is widespread and an overt understanding of the difference between classes (ideational/theoretical) and groups (phenomenological/empirical) evident, underlining the influence of Dunnell's thinking. Worth noting is that these approaches were embraced chiefly in only two South American countries – Argentina and Brazil – and largely independently of each other. Collectively, these works show that one of the most important contributions of *Systematics* has been as a bridge between archaeology and cognate fields – first and foremost palaeontology – dealing with challenges of classification and concerned with modelling macroevolutionary processes. The bridge-building that has followed from this has been very fruitful, reflected, for example, in the increasing number of archaeological presentations at meetings of the Cultural Evolution Society since the first one in 2017.

**Figure 3.**
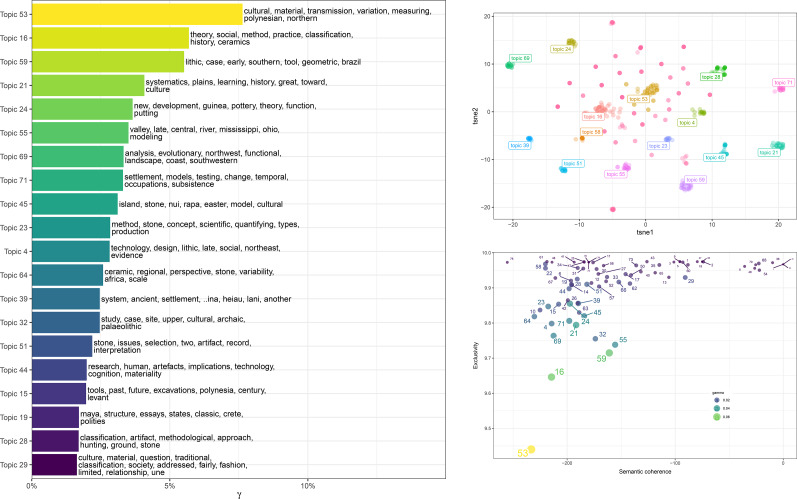
Left: Top 20 topics in titles of works citing ‘Systematics’. The gamma value indicates their overall abundance, and the topics are labelled with the most heavily weighted words in each topic. Right upper: clusters of citing works according to topic similarity, with clusters labelled by the most prominent topic. Clusters were computed using principal components analysis of the topic proportions in each citing work, then a *t*-distributed stochastic neighbour embedding (*t*-SNE) to reduce dimensionality, and density-based clustering to identify clusters. Each data point is one document. Right lower: plot of topics showing coherence and exclusivity metrics for each topic. Each data point is one topic.

The South American research stands out as a distinctly coherent and exclusive topic (Topic 59) in our topic model of titles of works citing *Systematics* (Figure 3). A topic model is an unsupervised classification of a collection of documents, in this case titles of works citing *Systematics*, that uses a probabilistic approach to generate mixtures of words that represent themes or topics in the collection (Roberts et al., 2014, 2016). This finds natural groups of topics similar to how clustering on numeric data finds groups of similar items. We generated a topic model with the widely used Latent Dirichlet Allocation method, which resulted in an optimal number of 76 topics. The topic model shows the persistence of the core themes of *Systematics* in the citing literature, with the most abundant and central topic (Topic 53) about the measurement of material culture variation and transmission of that variation. The topic model provides additional insights into the contribution of *Systematics* that are not evident in the word frequency analysis. For example, the archaeology of the central and southern Pacific Ocean stands out, and includes works on Polynesian fish hooks, fabrics and stone adzes. Rapa Nui has its own topic, representing the work of Carl Lipo and colleagues on the pottery, monuments and population dynamics of that island and the region. Lipo's work is thoroughly grounded in a concern for rigorous and explicit unit construction and innovative testing of archaeological systematics, noting that ‘systematics enables us to move beyond common sense’ (Lipo et al., 2021). We see a concern for the construction of analytical units in areas as diverse as the Palaeolithic of Africa, ceramics of the Maya and Papua New Guinea, and several regions of the USA.

The influence of *Systematics* has extended well beyond Dunnell's own primary study area of the North American Southeast. For example, the Portuguese translation published in 2007 has received 75 citations, and the Spanish translation 30. At face value these numbers seem low, but when we take into consideration that Binford's Portuguese translation of *In Pursuit of the past*, published in 1991, has similarly received a mere 31 citations, we can situate the influence of Dunnell's work in a more robust perspective. At the same time, in our data we see only a single work in an East or Southeast Asian language, a book chapter in Japanese surveying the literature on cultural phylogenetics, indicating limited attention to *Systematics* from researchers in the Eastern hemisphere. Could cultural differences in reasoning styles (Henrich et al., 2010) result in a diminished relevance for the philosophical content of *Systematics* outside of Western academic communities? Or perhaps it is simply that the book is quite difficult to read, as noted by reviewers when it was first published (‘confusion followed by grudging agreement’ is how Spaulding (1974, pp. 515–516) described his reaction to the book), and acknowledged by Dunnell in his foreword to the 2002 edition. The low readability of the book has probably limited the accessibility of its contents for readers whose first language is not English, and made it inherently less attractive as a pedagogical resource.

A second limitation of *Systematics* is that it failed to motivate the formation of a distinct research area and community of archaeological systematics. While biology has a Society of Systematic Biologists, and attendant journals such as *Systematic Biology*, *Systematics and Evolution* and *Systematics and Biodiversity*, archaeology currently has no equivalent focal points for discussions of systematics that transcends specific geographical and chronological concerns. *Systematics* was not a work that served as an engine for moving archaeologists together at scale to tackle questions about the formation of units of measurement and classification. Instead, these discussions typically happen deep within discrete and disconnected archaeological research communities, constrained by the culture and norms of those groups. The result of this fragmentation is a proliferation of bespoke classifications that lack applicability across cases. It also inhibits reproducibility, synthesis and the transfer of ideas and innovations across research communities, which may limit the sustainability of archaeology as a discipline.

Dunnell intended *Systematics* to establish a distinctive form of scientific archaeology, and it faced overwhelming competition from the programme advocated by the New Archaeology on the one hand, while also meeting resistance from deeply entrenched traditional approaches to classification. In addition, the volume also appeared just before personal computers were beginning to have a major impact on archaeological research practice (Aldenderfer, 1987; Clark & Stafford, 1982). The rigour advocated by Dunnell would have lent itself smoothly to the sorts of computational approaches now increasingly adopted in the discipline. Yet the approaches of the New Archaeologists dominated the literature in the decade after the publication of *Systematics*, thoroughly eclipsing it as a discipline-defining text. Nevertheless, what makes *Systematics* a classic contribution is that is provides archaeologists with the master key to release themselves from the ‘prison of de Mortillet’ (Shea, 2016, p. xvii), namely the inherited and entrenched analytical habits of research traditions dominated by economies of personal prestige (Gabriel de Mortillet, 1821–1898, was an archaeologist who published the first widely used classification of the Palaeolithic, much of which remains in use today). *Systematics* endures as a striking and precocious provocation to archaeologists to be transparent, rigorous, precise and deliberate about how we divide up and aggregate material culture and the measurements we take from it to describe and explain the human experience in the past. With archaeologists increasingly pursuing ambitious questions about evolutionary processes that require large-scale syntheses of disparate datasets (cf. Perreault, 2019), the message of *Systematics* will only become more relevant.

## Data Availability

The data that support the findings of this study are openly available in the Open Science Framework at http://doi.org/10.17605/OSF.IO/JBPFW.

## Research transparency and reproducibility

The data that support the findings of this study and the R code used to analyse and visualize the data are openly available in the Open Science Framework at http://doi.org/10.17605/OSF.IO/JBPFW. Readers may freely access these resources to reproduce all of the figures in the paper.

## Appendix: Colophon

This report was generated on 2022-04-21 08:43:35 using the following computational environment and dependencies:

The current Git commit details are:
